# Characteristics of Gut Microbial Profiles of Offshore Workers and Its Associations With Diet

**DOI:** 10.3389/fnut.2022.904927

**Published:** 2022-07-22

**Authors:** Chunhong Zhang, Dong Liang, Xiaoxue Li, Jun Liu, Mengya Fan, Mei Jing, Yifei Wang, Yu Zhang, Yiqun Fang, Dan Li

**Affiliations:** ^1^Navy Special Medical Center, Naval Medical University, Shanghai, China; ^2^Translational Medicine Research Center, Naval Medical University, Shanghai, China; ^3^Medical Innovation Research Division of Chinese General Hospital, Beijing, China; ^4^Independent Researcher, Shanghai, China

**Keywords:** gut microbiota, offshore workers, marine environment, *Holdemanella*, diet

## Abstract

The composition of gut microbiota is not a static state in humans but fluctuates in response to changes in environments, diet, and lifestyle factors. Here, we explored differences in gut microbiota between populations worked offshore and onshore and further studied microbiota-associated variables in offshore workers (OFWs). We investigated the gut microbiota of 168 healthy subjects (offshore: 145 and onshore: 23) using 16S rRNA sequencing. Our results indicated that the marine environment caused significant changes in intestinal microbial structure, which was mainly reflected in the increase in bacterial diversity, changes in composition, and the emergence of more specific bacteria in OFWs. In addition, characteristics of gut microbiota in OFWs were further explored, and the genus *Holdemanella* was considered a potential contributor to the stable state of health. Besides, some dietary factors, namely, duck, mutton, dairy products, and algae vegetables were identified as the gut microbial covariates in the OFWs cohort and were positively correlated with the genus *Holdemanella*. This suggests the positive intervention of diet on *Holdemanella*. Our data highlight, for the first time to our knowledge, that the marine geographical environment plays an important role in shaping the gut mycobiome composition. And diet could be considered as the targeted intervention that alters the composition of the microbiome to improve host health.

## Introduction

Alterations in the composition of the gut microbiome (i.e., dysbiosis) are associated with a broad range of human health disorders, namely, obesity, acne, rheumatoid arthritis, and inflammatory bowel disease (1–4). Lots of bacterial components have been revealed to involve in the pathogenesis of diseases by numerous disease-targeted microbiota studies, and can thus serve as bacterial biomarkers theoretically (5–7). For example, some microbial-derived markers were detected from human fecal samples, which may contribute to the noninvasive diagnosis of colorectal adenoma and could be targeted to suppress the colorectal cancer carcinogenesis (5). The influence of gut bacteria on host health, and the fact that the microbiome can be modified, have increased interest in developing microbiome-targeted therapies (8). However, there are still many challenges in applying microbiota research to the clinical practice, especially the characteristics of a healthy microbiome remain largely unknown, like the extent to which the gut microbiome is driven by genetic factors vs. environmental, lifestyle, or health-related factors that might be considered in microbiome-targeted therapies (9, 10).

The definition of a healthy microbiome cannot be precisely identified, because the composition of human gut microbiota is not a static state, and multiple factors could contribute to substantial differences across individuals, such as geographical environments, diet, lifestyle, and medication (9, 11, 12). For example, Lu et al. conducted a study on the gut microbiota characteristics in China and identified four microbic enterotypes from 2,678 healthy Chinese individuals, and found that geography explained the largest microbiota variation (9). This emphasized the influence of geography on gut microbiota composition and revealed the broad range of microbiota configurations in apparently healthy individuals. However, cohort studies on the effects of the marine geographic environment on the gut microbiota composition have been neglected, it remains to elucidate whether the marine geographic environment could affect the variation of gut microbiota.

Despite the great progress in microbiome research in recent years, population-based studies that integrate gut microbial profile with host demography, diet, lifestyle, and disease and medication usage remain scarce. These integrative phenotyping studies on large cohorts are essential for validating meaningful host–microbiota associations and identifying potential targets for microbiome-directed interventions.

Here, we provide an integrative analysis of the association between the gut bacteriome and host factors across 168 apparently healthy individuals in a large cross-sectional study. We identify factors belonging to demography, diet, lifestyle, and health status, that are associated with the composition of the gut bacteriome, namely, diversity, taxonomies, and inferred functional pathways. Stratifying individuals by the different geographical environments (offshore and onshore), we identify significant changes in gut microbial structure in offshore workers (OFWs) and emphasize the great influence of the marine geographic environment on the composition of gut bacteriome. Besides, characteristics of gut microbiota in OFWs were further explored, and the genus *Holdemanella* was considered a potential contributor to the stable state of health. Finally, we reveal the robust association of diet–bacteriome, this suggests potential opportunities for targeted interventions that alter the composition of the bacteriome to improve host health.

## Methods

### Cohort Description and Study Participant

The present descriptive cross-sectional study was conducted from December 2020 to January 2021. The 168 Chinese participants had no self-reported gastrointestinal tract disorder or any other acute/chronic/recurrent medical conditions (referred to as “healthy”). All recruited onshore workers (ONWs) or OFWs had lived locally for more than 1 year. Fecal samples were collected following a standardized procedure: the participants were informed of detailed instructions, collected samples by themselves, and stored samples in home freezers or iceboxes; samples were transported to the freezer at each sampling site within a day, and further to the research laboratory with cold-chain within 3 days; samples were then well homogenized, aliquoted, and stored at −80°C until further analyses. The metadata was collected *via* questionnaire, including demographic information [age, gender, height, weight, body mass index (BMI)], dietary information (taste preferences, food pairing, and cooking habits), lifestyle (alcohol intake, smoking, exercise frequency, wording h, sleep quality, and defecate frequency), and health status (common health problems, diseases, medication, and health products).

The study was approved by the Ethical Committee of Naval Medical University (Approval No. AF-HEC-018). Written informed consents were obtained from all participants.

### Analysis of Fecal Microbiota by 16S rRNA Sequencing

The DNA of total bacteria in fecal samples was extracted with a QIAamp Fast Stool DNA kit (Qiagen, Germany). The DNA samples were sent to Majorbio (Shanghai, China) for 16S rRNA relative quantitative sequencing. The general description of the 16S rRNA relative quantification sequencing was outlined in previous studies (13). Briefly, the V3–V4 region of bacteria 16S rRNA gene was chosen for amplification by PCR and sequenced on Illumina MiSeq platform using the 2 × 150 bp paired-end protocol. The primers used for amplification contain adaptors for MiSeq sequencing and dual-index barcodes so that the PCR products may be pooled and sequenced directly (14).

The paired-end sequences of each sample were exported from the MiSeq system for analysis in the FASTA format. The bioinformatic analysis of the sequences was performed in the VirtualBox of the Ubuntu Linux operating system, with different commands in the QIIME2 package (15). For the subsequent data analysis, we used R software (version 4.0.5) along with the Bioconductor Phyloseq (16) and MicrobiomeAnalyst (17) packages. To examine the functional capacity of participants' gut microbiome, we applied the PICRUSt (Phylogenetic Investigation of Communities by Reconstruction of Unobserved States) pipeline to predict the KEGG (Kyoto Encyclopedia of Genes and Genomes) orthology (KO) profile. The associations between phenotypic factors and the overall gut microbial composition were determined by PERMANOVA at the genus and KO levels (Bray–Curtis dissimilarities using the function adonis and the vegan R package, v2.5-6) in the OFWs cohort (18).

### Statistical Analysis

Descriptive analysis of the data consisted of calculating the means for quantitative variables and the relative frequencies (%) for categorical variables. The non-parametric test used was the Mann–Whitney U-test and the parametric Student's *t*-test. Statistical analyses were performed using IBM SPSS 19.0 version software. The differential abundance of taxa between groups was identified through the linear discriminant analysis (LDA) effect size (LEfSe) algorithm. Only taxon with LDA score >2 or *p* < 0.05 (Kruskal–Wallis test) was considered significantly enriched.

## Results

### Characteristics of Study Participants

We recruited 168 healthy volunteers from two different geographical environments, 23 individuals were ONWs from a normal terrestrial environment (control group), which included 100% males, with an average age of 22.74 ± 2.60 years, and an average BMI of 21.58 ± 1.39 kg/m^2^. And the other 145 were OFWs, which included 96.6% males, with an average age of 24.76 ± 3.83 years, and an average BMI of 23.13 ± 3.03 kg/m^2^ (Table 1).

**Table 1:** Items with statistical differences (*p* < 0.05).

	**Items**	**Onshore (*n* = 23)**	**Offshore (*n* = 145)**	***t*/*z***	***p***
Demography	Age	22.74 ± 2.60	24.76 ± 3.83	−3.21	<0.001
	Height (cm)	176.39 ± 5.11	173.54 ± 5.15	2.47	0.01
	BMI	21.58 ± 1.39	23.13 ± 3.03	−4.04	<0.001
Taste preferences	Light *n* (%)	19 (82.61%)	88 (60.69%)	−2.02	0.04
	Salty *n* (%)	2 (8.70%)	48 (33.10%)	−2.37	0.02
Cooking habits	Frying *n* (%)	9 (39.13%)	98 (67.59%)	−2.63	0.01
	Simmer *n* (%)	6 (26.09%)	77 (53.10%)	−2.40	0.02
Defecation frequency	<3 per week	2 (8.70%)	7 (4.83%)	−2.09	0.04
	3–7 per week	9 (39.13%)	31 (21.38%)		
	<3 per week	2 (8.70%)	7 (4.83%)		
Dietary habits	Eat fruit after meal *n* (%)	18 (73.26%)	82 (56.55%)	−1.96	0.05
Health status	Dizziness anemic *n* (%)	2 (8.70%)	2 (0.014%)	−2.13	0.03
	No health problems *n* (%)	18 (78.26%)	68 (46.90%)	−2.79	0.01

Statistical analysis found no significant differences in the vast majority of dietary variables and all the lifestyle variables between these two populations but noticed that the percentage of OFWs without any health problems was significantly lower than that of ONWs (46.90 vs. 78.26%, *p* = 0.01). Which suggested geographical environment, rather than diet or lifestyle, maybe a major factor in poor health among OFWs (Table 1).

### Gut Microbiota Diversity and Composition Differ Between ONWs and OFWs

**Figure 1:**
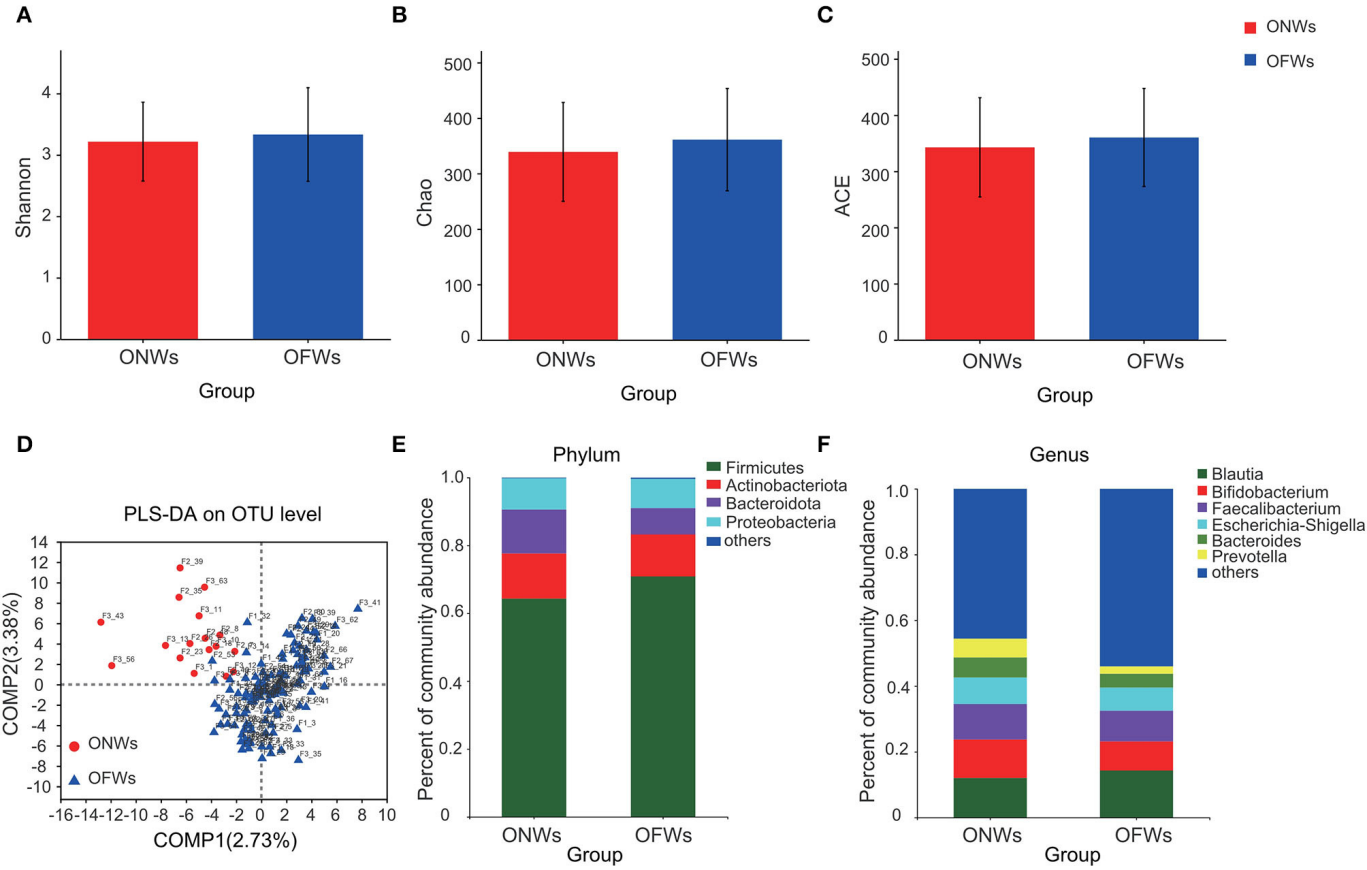
The diversity and relative abundances of the microbial community of ONWs and OFWs. Alpha-diversity sanalysis with Shannon **(A)**, Chao **(B)**, and ACE **(C)** index separately. **(D)** PLS-DA on OTU level. Relative abundance of the microbial community at phylum **(E)** and genus levels **(F)**. ONWs, onshore workers; OFWs, offshore workers.

Diversity analysis showed a trend of increase in bacterial diversity in OFWs (Figures 1A–C). PLS-DA analysis indicated that the gut microbiota in OFWs was distinctly dissimilar to that in ONWs (Figure 1D), which indicated a great change in gut microbiota compositions influenced by the marine geographical environment.

The dominant phyla of gut microbiota were *Firmicutes, Actinobacteriota, Bacteroidota*, and *Proteobacteria* in these two populations (Figure 1E). Compared with the ONWs, the OFWs had a much higher proportion of *Firmicutes* (64.33 vs. 70.88%), but a lower proportion of *Bacteroidota* (12.94 vs. 7.78%). Similar results were observed at the genus level, *Blautia* (phylum *Firmicutes*) increased in OFWs, while *Bacteroides* and *Prevotella* (phylum *Bacteroidota*) decreased (Figure 1F).

### Enriched Microbes in ONWs and OFWs

**Figure 2:**
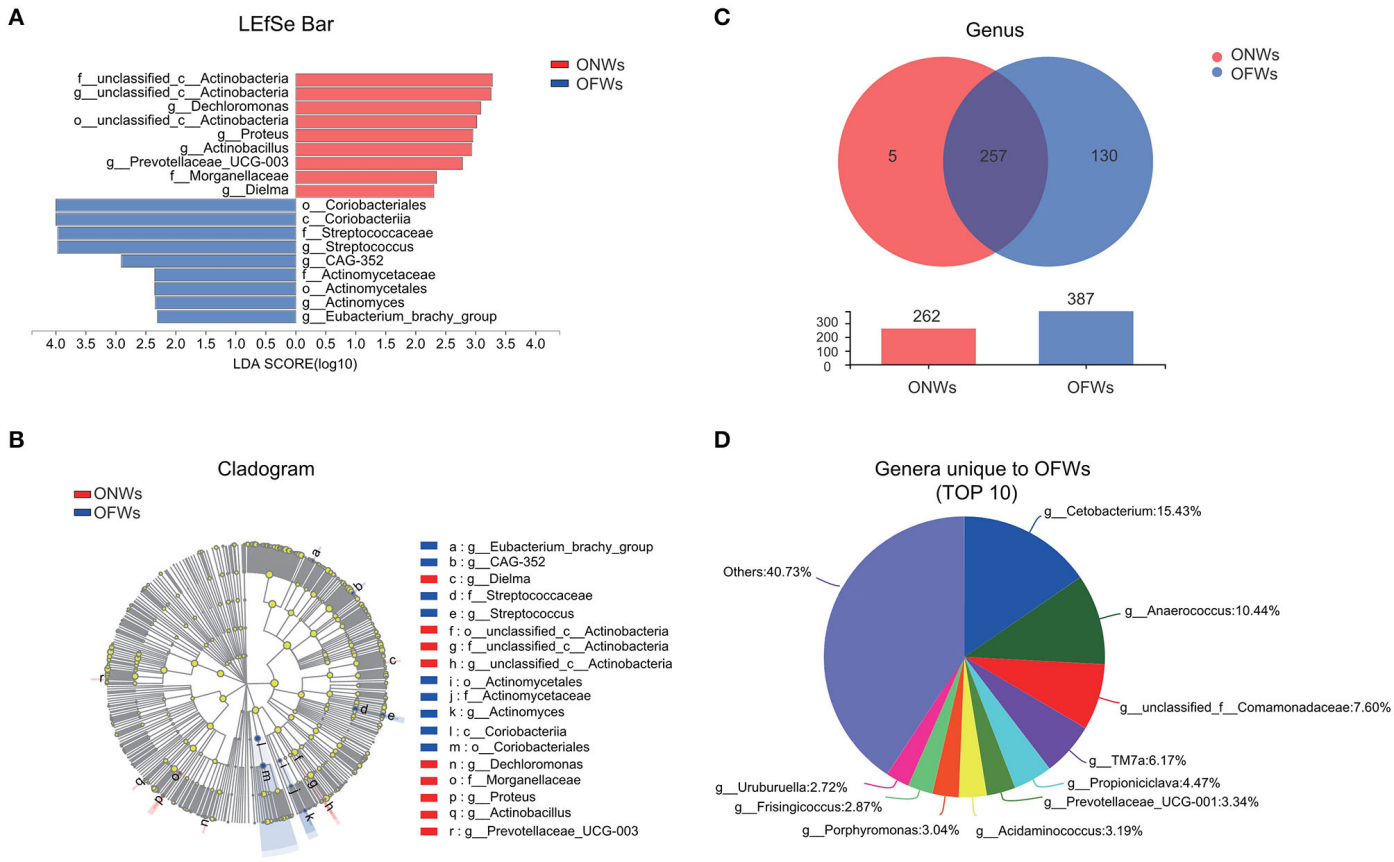
The microbiota profile differs between ONWs and OFWs. **(A)** Histogram of the linear discriminant analysis (LDA) scores for differentially abundant taxonomic features between ONWs and OFWs. **(B)** Cladogram of the microbial taxa associated with ONWs and OFWs. Significance obtained by LDA score >2. **(C)** The Venn diagram compares and contrasts the number of taxa at genus levels of ONWs and OFWs. **(D)** The top 10 genera are unique to OFWs. ONWs, onshore workers; OFWs, offshore workers.

To further investigate the impact of marine geographical environment on gut microbiota, LEfSe analysis was conducted to identify discriminative taxa, and 9 taxa were identified in both the groups (Figures 2A,B). Besides, there were more unique genera in OFWs than ONWs (130 vs. 5) (Figure 2C), this result corroborated the above findings of the increased diversity of gut microbiota in OFWs. Specifically, the genera unique to OFWs mainly include *Cetobacterium* (15.43%), *Anaerococcus* (10.44%), *unclassified_f_Comamonadaceae* (7.60%), *TM7a* (6.17%), and *Propioniciclava* (4.47%) (Figure 2D). At the same time, OFWs also lost five genera, mainly including *Proteus* (84.32%) and *Prevotellaceae_UCG-003* (13.94%), while these two genera enriched significantly in ONWs as well (LDA>2) (Supplementary Figure S1; Figure 2B).

Taken together, these results revealed a remarkable bacteriome structural shift in OFWs, indicating the important influence of the marine geographical environment on gut bacteriome.

### *Holdemanella* Contribute to the Health of OFWs

**Figure 3:**
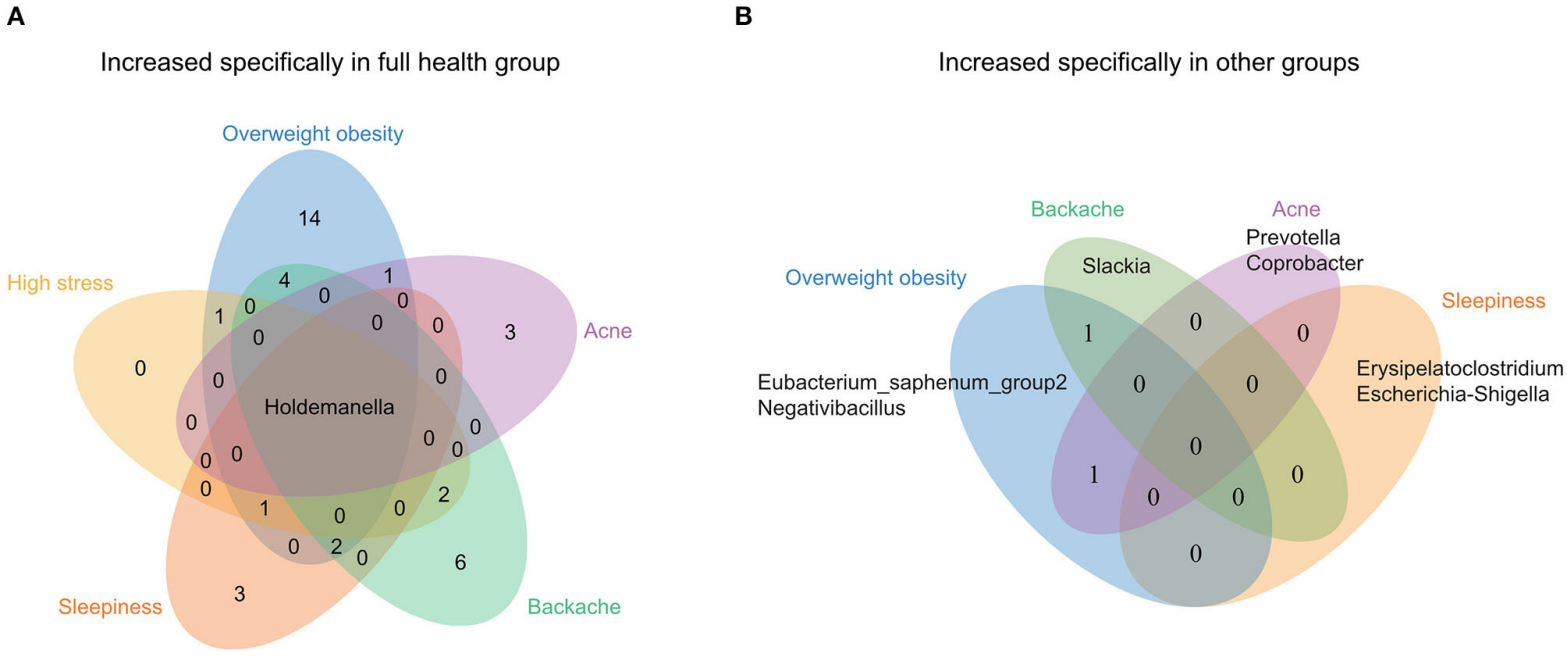
Venn diagram of distinct genera identified by LEfSe analysis among each of the population groups in the OFWs cohort. **(A)** Distinct genera in the Full health group. **(B)** Distinct genera in the other groups (Overweight obesity, High stress, Sleepiness, Backache, and Acne).

To further identify the characteristics of gut microbiota of OFWs, the populations were grouped by their health status according to the questionnaire results, and 6 groups (Overweight obesity, High stress, Sleepiness, Backache, Acne, and Full health) that contained more than 15 individuals without antibiotic use records were picked for further research. LEfSe analysis was conducted to identify discriminative taxa between the Full health group and other groups (Supplementary Figure S2). Notable, several unique genera were found. Among the enriched microbes in the Full health group, *Holdemanella* was the only genus that increased significantly compared with the other groups (Figure 3A), which suggested *Holdemanella* may be the potential contributor to the stable state of health. Besides, we found that all the other groups except High stress had unique genera which were increased specifically compared with the Full health group (Figure 3B). For example, genera *Erysipelatoclostridium* and *Escherichia-Shigella* increased specifically in the Sleepiness group.

### Identification of the Gut Microbial Covariates in OFWs Cohort

**Figure 4:**
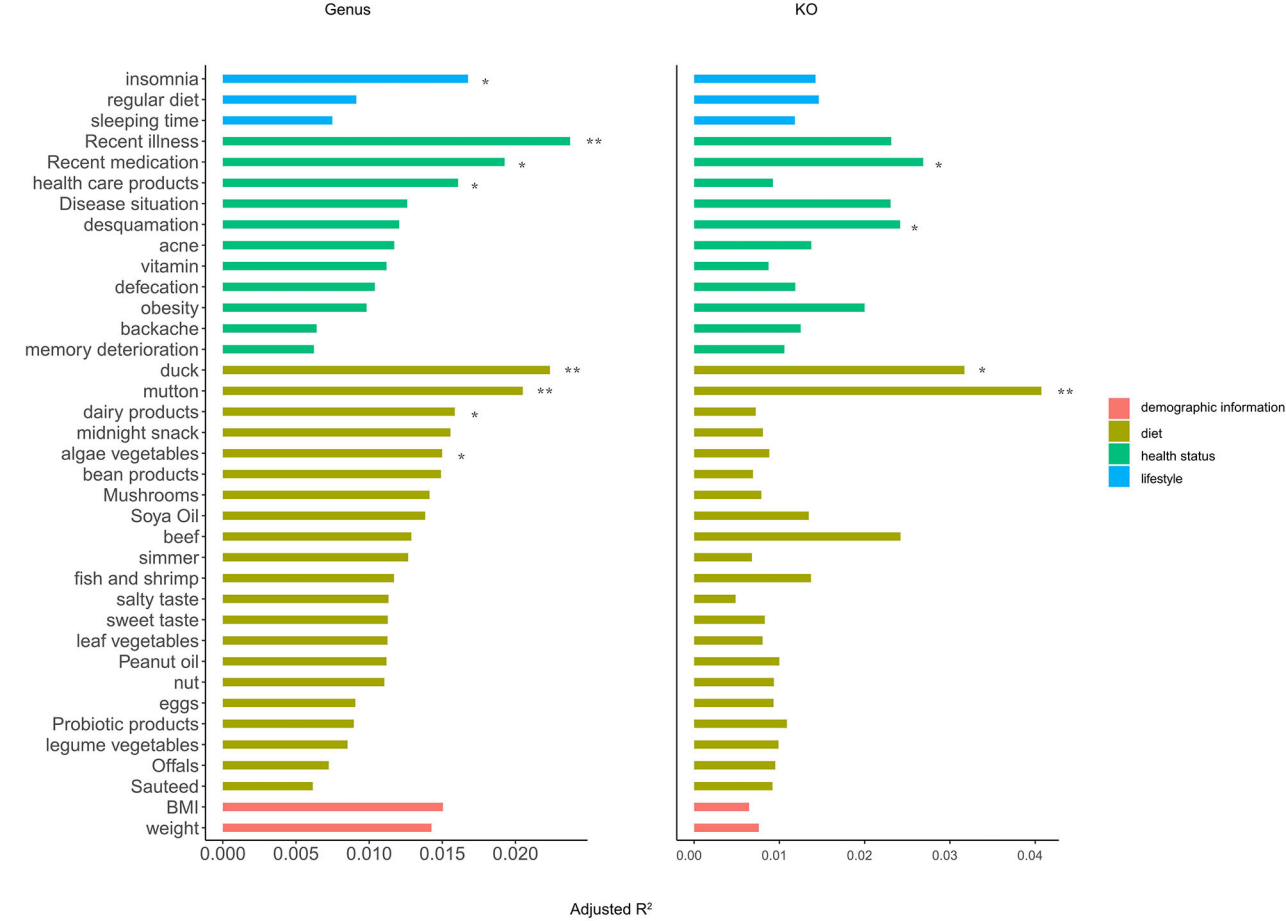
Identification of the gut microbial covariates in the OFWs cohort. Horizontal bars showing the amount of inferred variance (adjusted *R*^2^) explained by each identified covariate as determined by PERMANOVA with Bray–Curtis dissimilarities at the genus (left) and KO (right) levels. The categories are color-coded and ranked by the highest explained variation in the respective category. Only statistically significant covariates with an adjusted *p* < 0.05 using the Benjamini–Hochberg (BH) method are shown. **BH-adjusted *p* < *0.01*; *BH-adjusted *p* < *0.05*.

In the entire OFWs cohort, nine factors were consistently identified as significant microbiota covariates at the genus and KO level (PERMANOVA, adjusted *p* < 0.05; Figure 4). Compared to other factors, diet habits, namely, regularly eating duck, mutton, dairy products, and algae vegetables explained most microbial variance in the OFWs cohort, followed by factors about health status such as recent illness, recent medication, and regularly eating healthcare products. Lifestyle factors associated with insomnia also showed significant impacts on the gut microbiota. In addition, BMI and weight might be the potential influencing factors of gut microbiota.

These results indicate that diet (regularly eating duck, mutton, dairy products, and algae vegetables) could be used as the main means to interfere with the composition of intestinal microbes, and individual health status (illness, medication, and healthcare products) and unhealthy lifestyle (insomnia) also have a greater impact on gut microbiota.

### Host Factors Associated With Gut Bacteriome in OFWs Cohort

**Figure 5:**
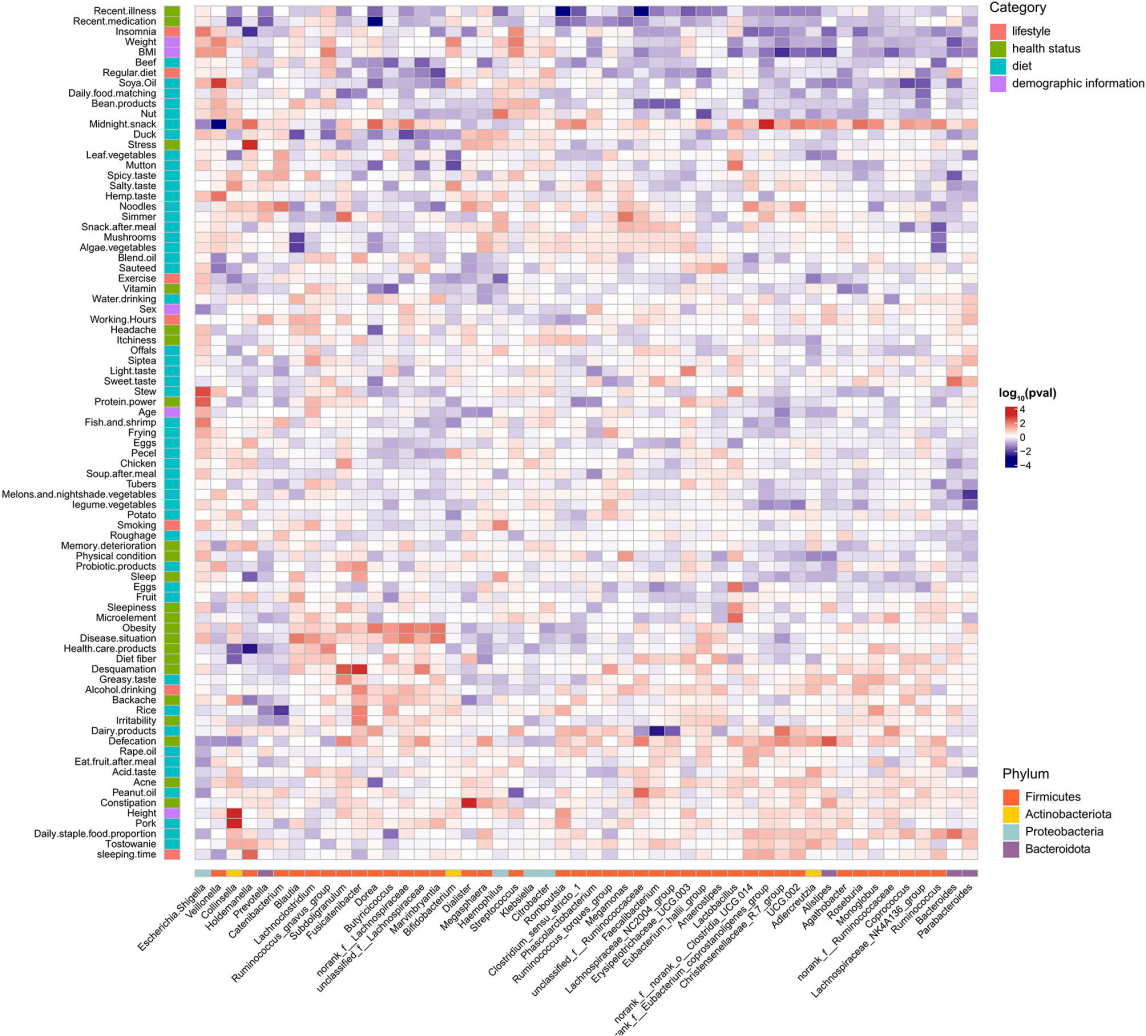
Associations between microbial genera and multiple factors. Shown is a heatmap of the microbial genera (x-axis) that were found to be significantly associated with different factors (y-axis) using generalized linear models adjusted for confounding factors. The significant heatmap cells are represented by the direction of association (indicated by color, e.g., red is positively associated). Each factor is colored by the category to which it belongs, and each genus is colored by the phylum to which it belongs.

**Figure 6:**
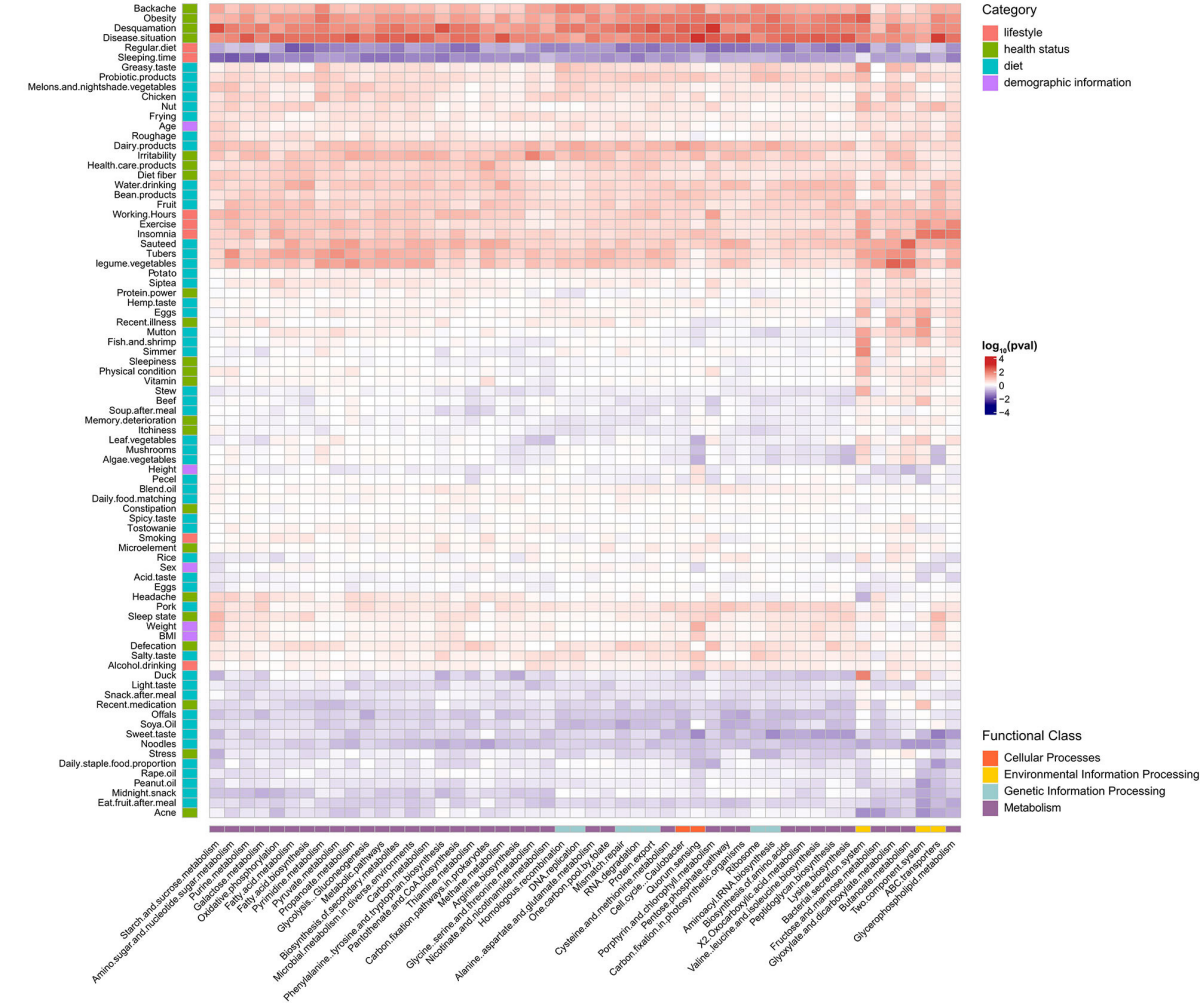
Associations between microbial pathways and multiple factors. Shown is a heatmap of the microbial pathways (x-axis) that were found to be significantly associated with different factors (y-axis) using generalized linear models adjusted for confounding factors. The significant heatmap cells are represented by the direction of association (indicated by color, e.g., red is positively associated). Each factor is colored by the category to which it belongs, and each microbial pathways is colored by the functional class to which it belongs.

We next explored the relationship between host factors (*n* = 83) and individual genera and inferred functional pathways (Figures 5, 6). Weak correlations between host factors and the abundance of gut microbiota and pathways were identified. We found that the genus *Holdemanella* was negatively correlated with factors about health statuses such as BMI, Sleepiness, Backache, and Acne, and only Backache with a significant correlation (*q* = 0.028). Moreover, genus *Holdemanella* positively correlated with diet habits factors, namely, regularly eating duck, mutton, dairy products, and algae vegetable (Figure 5). Of note, the bacterial secretion system pathway was positively associated with these diet habits factors, among which duck and mutton with a significant correlation (*q* < 0.05) (Figure 6). Taken together, these results support the positive health effects of *Holdemanella* and demonstrate the positive intervention of diet on *Holdemanella* and the bacterial secretion system pathway.

## Discussion

We collected stool samples of individuals from different geographical environments (onshore and offshore). Based on high throughput sequencing data, we analyzed the composition of gut bacteriome and the potential influence of 83 host factors. We found a remarkable bacteriome structural shift in OFWs compared to ONWs. Moreover, we further detected the characteristics of gut microbiota of OFWs, and identified the covariates of gut microbiota. It is worth noting that OFWs cohort was the first time to be studied. The great effects of the marine geographical environment were reinforced and the changes in gut microbiota following the marine geographical environment were studied first.

It is well acknowledged that the important role of the geographical environment in shaping human gut microbiota (19, 20), but it remains to elucidate whether the maritime environment could affect the variation of gut microbiota. In this study, we found a trend of increase in bacterial diversity in OFWs, and a large number of specific genera (*n* = 130) were identified (e.g., *Cetobacterium, Anaerococcus, unclassified_f_Comamonadaceae, TM7a*, and *Propioniciclava*). It should be noted that the genus *Cetobacterium* accounted for the highest proportion (15.43%) among these emerging bacteria. Considerable works indicate that the gut community of fish is characterized by a large proportion of *Cetobacterium*, a genus previously linked to carnivorous species (21–23). And there are some factors driving the establishment of this genus in the gut, namely, trophic level, salinity, and vitamin B12 availability (22, 24, 25). Considering the long-term offshore operations of OFWs and the adaptation of gut bacteriome to the environment (26), it is not difficult to comprehend the emergence of convergent signals in intestinal microbial composition in humans and fish.

With the development of next-generation sequencing technology, researchers have gained a better understanding of the composition of human gut microbiomes, but we are still struggling to pin down which components are essential to human health (27). One of the concerns is the statistical connections between the microbiome and health or disease are difficult to establish due to a lack of data (27). In this study, connections between host factors and bacteriome genera were explored. *Holdemanella* was significantly enriched in the full healthy group of OFWs cohort, and correlation analysis showed that it was negatively correlated with some common health problems (Sleepiness, Backache, and Acne), which suggests that the genus *Holdemanella* may be the potential contributor to the stable state of health (referred to as “healthy microbe”). Previous studies have already demonstrated the health benefits of *Holdemanella* (28–30). It was reported that *Holdemanella* was one of the dominant abundance genera of healthy controls compared with patients who underwent irritable bowel syndrome (31), which is similar to our research. Furthermore, *Holdemanella biformis* (*H. biformis*), belonging to the genus *Holdemanella* has been considered an anti-inflammatory bacteria (30). Pujo et al. found that *H. biformis* could produce a high concentration of long-chain fatty acid hydroxylated on the third carbon to modulate inflammation, which in turn the probiotics to play therapeutic effect in inflammatory bowel diseases (30). However, opposite results were also reported in previous studies. One cohort study reported that the genus *Holdemanella* was enriched in Tibetan Highlanders suffering from coronary artery disease compared with healthy Tibetan Highlanders and Han coronary artery disease patients living at high and low altitudes (32). Several other studies have also found that *Holdemanella* was significantly increased in patients suffering from autism spectrum disorder, colorectal cancer, or chronic kidney disease (33–35). The differences in the results reaffirmed the challenge of distinguishing a healthy microbiome from an unhealthy one. Potentially huge differences between the gut microbiomes of apparently healthy individuals could partly explain the challenge (36, 37). These differences are generated by the combined effect of genetic, geographical, dietary, and lifestyle factors (12, 38). In practice, this means considering not only the biomarkers of health or risk of disease but also the individuals' geographic location, lifestyle, and other factors before determining whether an individual is relatively healthy or at increased risk of developing disorders. Thus, genus *Holdemanella* may be considered a healthly microbe just in people working in an offshore environment for a long time, it might not be healthy in another context.

It is well acknowledged that diet alters gut microbial composition and metabolism. In our study, duck, mutton, dairy products, and algae vegetables were identified as the gut microbial covariates and showed a positive correlation with *Holdemanella*. Similar results were also demonstrated in previous studies. It was reported that *Holdemanella* was positively correlated with a fermented dairy product, carbohydrate, and fiber intake (32). In addition, meat consumption pattern was also an important factor, Lin et al. found that *Holdemanella* was enriched in populations with a preference for white meat (e.g., poultry and aquatic product) consumption (39). These findings suggested that directed dietary interventions could be considered as a potential strategy to improve host health by enriching the beneficial bacteria (40). In addition, we predicted KEGG pathways based on functional profiles using PICRUSt. We found that the bacterial secretion system was significantly positively associated with duck and mutton. The bacterial secretion system can deliver effector proteins, namely, nucleic acids, nucleotides, proteins, and lipids, into the extracellular milieu, thereby conferring a competitive advantage to the bacterium and promoting its survival (41, 42). Bacterial type VI secretion system, for instance, could deliver antibacterial effectors resulting in microbial antagonism to inhibit the proliferation of competitors in the gut (43). However, the interbacterial antagonism does not imply the dysbiosis of the gut, when considered in the broader ecological context of the microbiota and symbiosis with the host, the bacterial secretion system may also promote the symbiotic relationship with the host by enabling metabolic cooperation (44, 45). Our findings, in combination with the previous literature, suggested that dietary factors including duck and mutton might activate the bacterial secretion system, which in turn promotes the proliferation of beneficial bacteria, furthering the stability of gut ecosystems. However, the internal relationships among diet, microbiota, and bacterial secretion system require further validation with multiomics integrative studies.

We acknowledge several limitations of this study. First, we are limited by taxonomic classifications based on 16S rRNA amplicon profiling rather than comprehensive shotgun metagenomic sequencing data. Second, as the OFWs cohort is volunteer-based and the particularity of occupation, our cohort suffers from sample limitation and gender unbalance. Also, the study might attract people who are more interested in their health. Lastly, for some of the diseases, a relatively low number of cases were available, which could mean a low statistical power for detecting an effect of the microbiome. Therefore, the associations identified in the current study should be carefully interpreted and require further investigation using larger sample sizes. We acknowledge that some findings of our study are speculative and correlative, and future studies are warranted to unravel the cause-vs-consequence relationship between the gut bacteriome and host factors, as well as the downstream mechanistic aspect.

In summary, we have found the “healthy microbe” in OFWs and the significant microbiota covariates. It is possible that interventional strategies for the health of offshore people in the future may include therapeutic interventions designed to affect the bacteriome such as probiotics, fecal transplantation, and directed dietary or lifestyle interventions, although more work is required. In addition, characterization of the gut bacteriome in OFWs may provide biomarkers for health prediction and could theoretically be an avenue to prevent health problems in offshore people.

## Data Availability Statement

The datasets presented in this article are not readily available because the particularity of the participants. Requests to access the datasets should be directed to CZ.

## Ethics Statement

The studies involving human participants were reviewed and approved by Committee on Ethics of Medicine, Navy Medical University, PLA. The patients/participants provided their written informed consent to participate in this study.

## Author Contributions

CZ, YF, and DLi: conceptualization, supervision, and project administration and funding acquisition. CZ, DLia, and DLi: methodology. CZ and DLia: software, formal analysis, visualization, and writing of the original draft preparation. CZ, DLia, XL, JL, MF, MJ, YW, YZ, YF, and DLi: validation. CZ, DLia, YF, and DLi: writing, reviewing, and editing. All authors contributed to the article and approved the submitted version.

## Funding

This research was funded by the Construction of key military disciplines (2020SZ20-3) and the Deep Blue Talent Project of Naval Medical University (21TPSL0601).

## Conflict of Interest

The authors declare that the research was conducted in the absence of any commercial or financial relationships that could be construed as a potential conflict of interest.

## Publisher's Note

All claims expressed in this article are solely those of the authors and do not necessarily represent those of their affiliated organizations, or those of the publisher, the editors and the reviewers. Any product that may be evaluated in this article, or claim that may be made by its manufacturer, is not guaranteed or endorsed by the publisher.

## Supplementary Material

Supplementary Figure S1 Genera unique to OFWs. OFWs, offshore workers.

Supplementary Figure S2 Histogram of the linear discriminant analysis (LDA) scores for differentially abundant taxonomic features between Full health group and the other groups (Overweight obesity, High stress, Sleepiness, Backache and Acne). Significance obtained by LDA score >2.
