# Supplementary figures and images for: Characteristics of Gut Microbial Profiles of Offshore Workers and Its Associations With Diet
Source: Front Nutr. 2022 Jul 22;9:904927. doi: 10.3389/fnut.2022.904927 (PMC9354959; doi:10.3389/fnut.2022.904927)

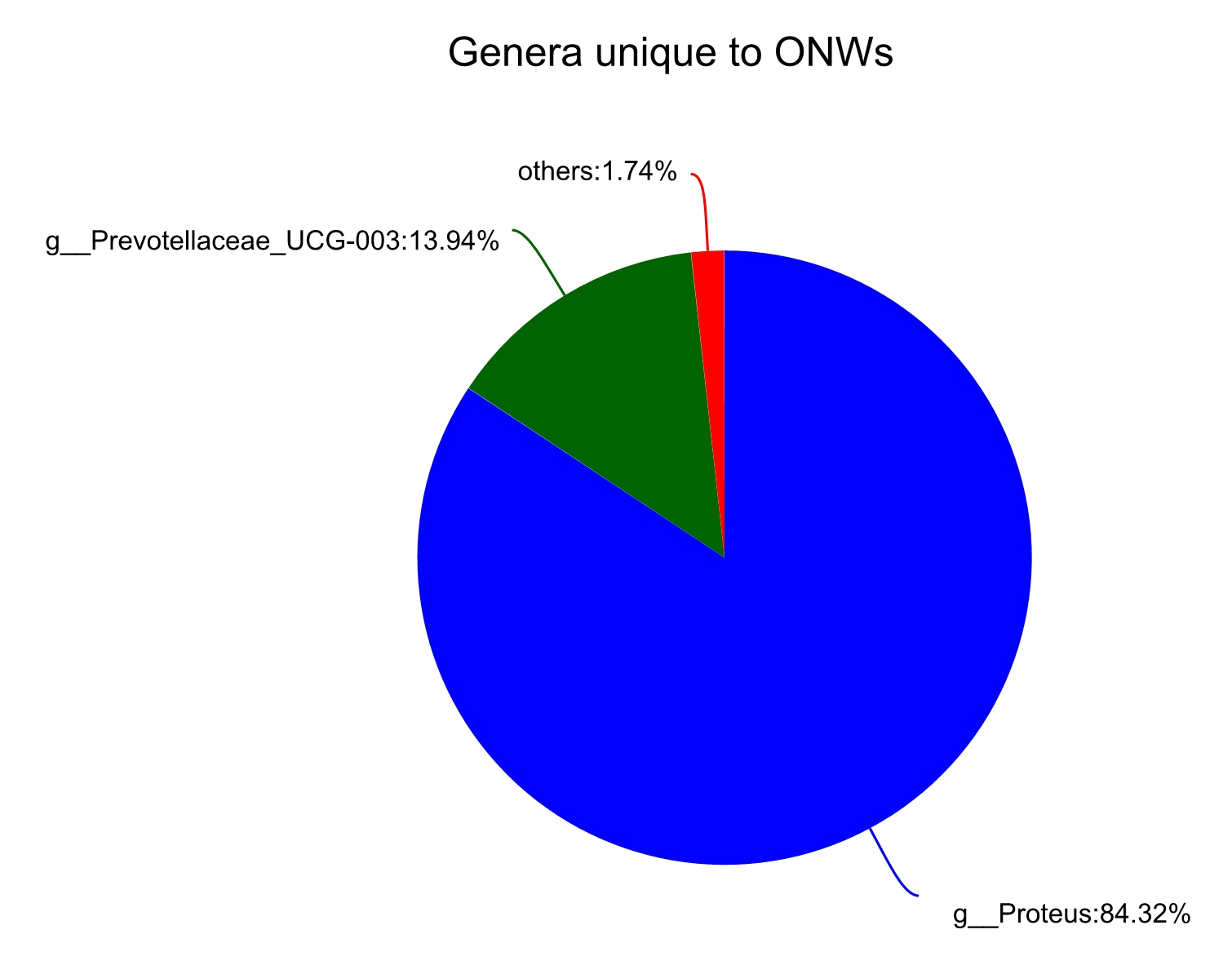

Supplement: Supplementary Figure S1 — Genera unique to OFWs. OFWs, offshore workers. [file Image_1.JPEG]

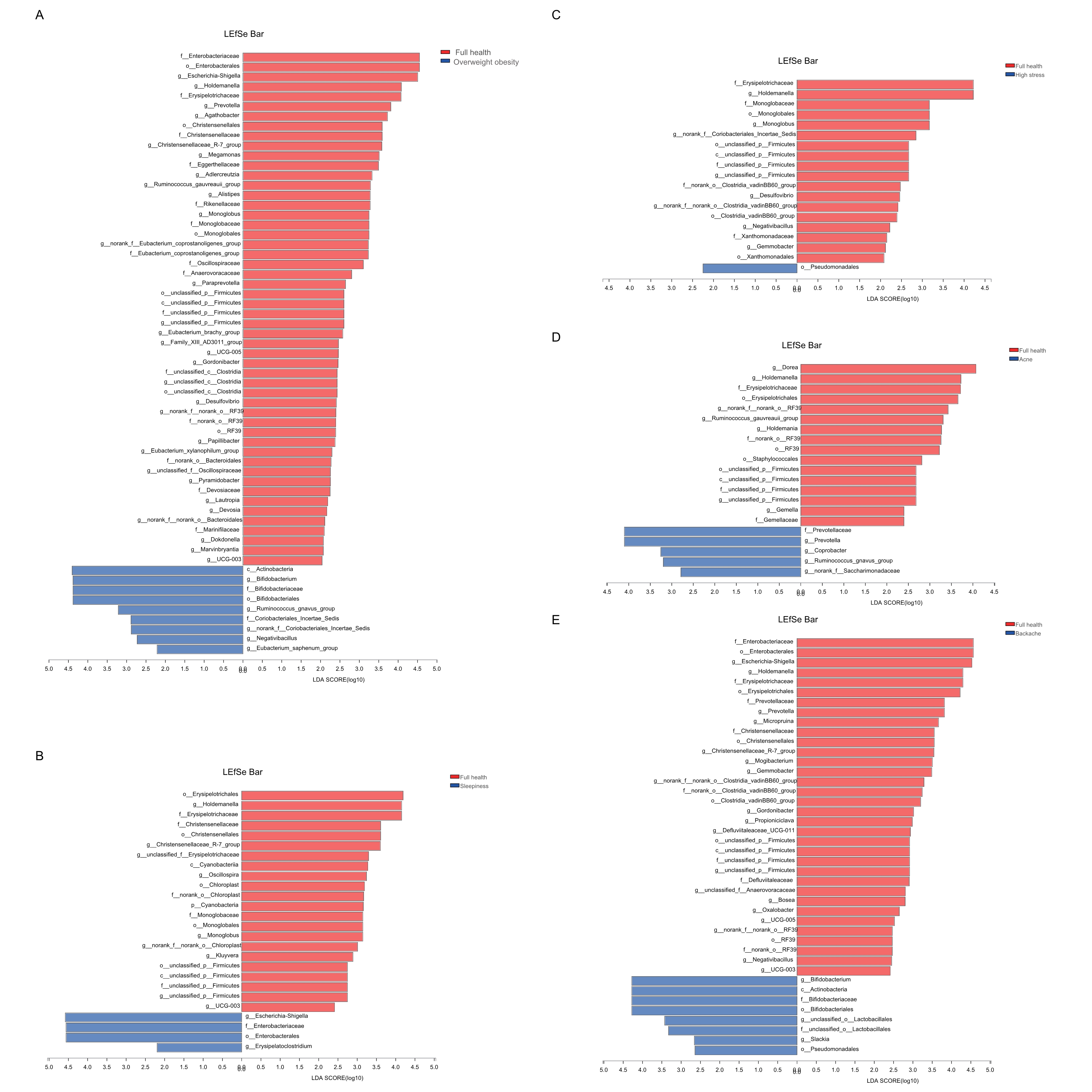

Supplement: Supplementary Figure S2 — Histogram of the linear discriminant analysis (LDA) scores for differentially abundant taxonomic features between Full health group and the other groups (Overweight obesity, High stress, Sleepiness, Backache and Acne). Significance obtained by LDA score >2. [file Image_2.JPEG]
